# Food Addiction: An Evolving Nonlinear Science

**DOI:** 10.3390/nu6115370

**Published:** 2014-11-21

**Authors:** Richard Shriner, Mark Gold

**Affiliations:** Department of Psychiatry, University of Florida, Box 100183, Gainesville, FL 32610-0183, USA; E-Mail: drmarkgold@gmail.com

**Keywords:** obesity, food addiction, diabesity, type 2 diabetes, bariatrics, nutrition, nonlinear, IGS-IT (Information Gathering and Sharing-Information Technology), IGS-Flow Game, IGS-rooms

## Abstract

The purpose of this review is to familiarize readers with the role that addiction plays in the formation and treatment of obesity, type 2 diabetes and disorders of eating. We will outline several useful models that integrate metabolism, addiction, and human relationship adaptations to eating. A special effort will be made to demonstrate how the use of simple and straightforward nonlinear models can and are being used to improve our knowledge and treatment of patients suffering from nutritional pathology. Moving forward, the reader should be able to incorporate some of the findings in this review into their own practice, research, teaching efforts or other interests in the fields of nutrition, diabetes, and/or bariatric (weight) management.

## 1. Introduction

“Leave your drugs in the chemist’s pot if you can cure the patient with food.”—Hippocrates, 420 B.C.

Even before Francine Kaufman coined the term *diabesity* [1], researchers and clinical investigators had been made painfully aware of the clinical colocalization of obesity and diabetes. Now, because of its global footprint, perhaps the term *diaglobesity* should be added to our vocabulary. Sullivan recently reported that both diabetes and prediabetes prevalence rates are sharply on the rise [2]. Hwang, Bais, Sun and Chen were able to show in a five year follow up study of 1547 healthy obese patients, many suffered from a higher risk of hypertension, type 2 diabetes (T2DM) and metabolic syndrome than non-obese controls [3]. Greevenbroek, Schalkwijk and Stehouwer give an excellent review of the entire inflammatory diabesity process leading from obesity to insulin resistance to the emergence of T2DM [4]. Farag and Gaballa argue that 14% (for diabetes) and 7% (for obesity) of the entire U.S. Health Care Budget is being spent on diabesity patients with a 2030 projected cost of $490 billion [5]. These same investigators project by 2025 that China and India will be spending 40% of their entire health care budget on diabesity as well. Weck, Bornstein, Barthel and Bluher have reported as high as an 80% relapse rate (*i.e.*, failure in dieting) in obese patients [6]. Finally, Wadden and Phelan point out that 30%–50% of obese patients and 20% of nonobese patients underestimate their caloric intake [7].

Given the above, it would appear that: (1) obesity is associated with a host of other illnesses that in combination exact tremendous impact on morbidity and mortality worldwide; (2) obesity and diabesity are on the rise; (3) obesity and diabesity both qualify as pro-inflammatory disease states; (4) attempts at curtailing obesity through dieting largely fail and such patients are subject to frequent “relapse” (*i.e.*, they regain their original weight); and (5) almost one half of obese patients seem to inaccurately report or remember the real amount of caloric intake they consume. Since 2013, obesity has been recognized by the American Medical Association [8] to be a disease.

In contrast to the treatment of a disease such as diplococcal pneumonia, obesity treatment is either ineffective or achieves a less than anticipated rate of success. Unlike most cases of diplococcal pneumonia (*i.e.*, in non-immunocompromised hosts), obesity is a recurring disease that is characterized by frequent relapses. Equally important, as Wadden and Phelan clarify [7], obesity is also a disease that appears capable of influencing the objectivity and accuracy of how patients report the exact amount of abused substance they ingest, not unlike that seen in alcoholics and other patients suffering from addictive disorders.

It is for all of the above reasons that many bariatric (meaning weight affiliated) clinical and bench lab investigators are now turning to addiction modeling to better understand obesity and other forms of disordered eating. Over the last decade, there have been significant discoveries made in the science of food addiction. We will outline some of these within this review. We will also introduce three separate but related models for weight science and obesity care. These models employ nonlinear principles to illuminate the possible contribution of food addiction to the study of obesity and improve the accuracy and usefulness of our current linear models for treatment.

Finally, in reference to the Hippocrates quote cited above, it would seem oxymoronic to assume that the cause of an ailment simultaneously forms the substance of its cure, but with obesity, diabesity and food addiction, this is decidedly the case. Shriner [9,10] and others [11,12] have argued that food/macronutrient prescription forms the first line therapy to treat most cases of obesity and diabesity. This does not dismiss the importance of bariatric pharmacology or surgery, but these are second line backups and should never be a substitute for more basic primary nutritional care. As the reader of this paper will discover, it becomes increasingly obvious that the aberrant use of food in the form of food addiction unlocks and explains, at least in part, why many diets fail over time. Simple pharmacology or surgery cannot recalibrate this abnormality. As you often hear from bariatric surgical patients, “It’s still me, Doc, they didn’t operate on my mind, just my stomach.” This is why the treatment of obesity and diabesity requires a multimodal approach; their origins are multifactorial. With this in mind, we will direct our discussion in a way most useful to the entire spectrum of bariatric clinicians: dieticians, bariatric physicians, nurses, psychologists and counselors, exercise therapists and the rest of those clinicians and administrators who are integral to the entire bariatric treatment team.

## 2. Food Addiction’s Early Roots and Emerging Models

Food addiction is not a new science, especially given the early and groundbreaking investigations carried out by Gold [13,14,15,16,17,18] and others [19,20], which extend well over a twenty year period. Food addiction, and the bench lab neuroscience that supports it, has undergone a considerable evolution over this same period. This includes the invention of lab animal models for both addiction and disorders of eating. It also includes recent advances in the study of addictive metabolism, delineating the key neurochemistry that fuels addiction and the influence that food has on addictive anatomical channels in the brain revealed by neuroimaging [21,22]. This evolution has and will continue to have a tremendous impact on bariatric, nutritional and diabetic science, especially in terms of aiding in the discovery of more potent dietary, pharmaceutical and surgical interventions for the treatment of obesity and diabesity.

Food addiction neuroscience, as this paper will illuminate, is revolutionizing our understanding of how obesity evolves and sustains itself through time. This may include how weight is either gained or lost in specific eating disorders such as anorexia, bulimia, binge eating,* etc.* Equally important, food addiction may constitute the missing link that helps to explain the often unpredictable and nonlinear interplay between select macronutrients (e.g., high sugar and high sugar/high fat) and maladaptive psychological adaptations to food. We will introduce three important models that serve to integrate food addiction with dietary/nutritional science and the study of psychological adjustments that underlie disorders of eating. The first model (a Tripartite Model) incorporates metabolic, addictive and behavioral (*i.e.*, relationship) drivers of weight. The second model (a stress/weight matrix called SWEAM, which stands for: Stress, Weight, Eubaric, Allobaric and Matrix) helps to illustrate how food addiction, through the processes of cueing and craving, has a significant impact on obesity and other disorders of eating. The last model is a simple but powerful neurochemical map for weight. It diagrams how key macronutrients create the thermic energy drivers that stimulate gut peptides, neurometabolic transmitters and endocrines which then travel to the brain to stimulate either net weight gain or loss.

We will now discuss how the modeling of obesity and food addiction, using nonlinear (*versus* traditional linear) models, sheds an entirely new light on why many diets fail, or at least fail to live up to their original expectations. Using nonlinear science, we will show how the three models we have just identified may assist in the creation of more effective obesity and diabesity treatments.

## 3. Bariatric Models: Linear* vs.* Nonlinear

Linear models in physics, mathematics, biology,* etc.*, are ones that assume relatively stable, consistent, and predictable interactions (sometimes referred to as static* versus* dynamic) between components that constitute processes in nature. For example, Newtonian physics states that under ideal circumstances, two cannon balls of equal weight (one simply dropped, the other fired from a cannon, but both from the same initial location) will fall to earth at exactly the same time. This means that the Y component of acceleration of both cannon balls (gravity at 9.8 m/s) and the X component of acceleration of the fired cannon ball (determined by the energy transferred at the time of discharge) are *independent* of each another. [23] Therefore, one can calculate the exact trajectory of the fired cannon ball, as well as when both shot and dropped cannon balls will simultaneously hit the ground.

Using an obesity (dietary) science analogy, let us assume both cannon balls weigh the same (*i.e.*, they have the same caloric content). We will call this factor “energy intake.” Let us say we can also calculate the exact speed that the fired cannon ball will achieve. We will call this factor “energy expended.” Now, with these two components in mind, using linear modeling (and the First Law of Thermodynamics to complete our nutrition analogy) we should be able to calculate the exact position where and the time when the cannon balls land. This last factor we will call “net weight, gained or lost.” We know this as the classic linear thermodynamic equation, net weight = energy (caloric) intake − energy expended (exercise, thermic effect of food, metabolic rate,* etc.*).

Nonlinear models are much different. Specifically, in a nonlinear world, a calorie is not always the same calorie. Hence, calories derived from a gram of high fructose corn syrup (as proposed by Lustig [12]) may not affect the system in the same way a gram of table sugar will. In fact, the former (high fructose corn syrup) may lead to more weight gain. Equally vexing, using our acceleration of gravity analogy for energy expended, this energy may vary based on changing external and internal factors. For example, an animal living under high CRF (corticotropin-releasing factor) tone may expend a different amount of energy per calorie ingested than one living under low CRF tone. In other words, sometimes the same patient ingesting the same amount of calories and arguably expending the same amount of energy (e.g., running a mile a day), will lose weight, at other times they will break even and at still others, they may actually gain weight! Using our cannon ball analogy, the cannon ball is falling at different rates at different times! Those of us who care for obese and eating disorder patients see this sort of non-linear deviation in actual and pre-calculated patient weight quite frequently. In other words, weight gain or loss is dynamic (not static), at times unpredictable and chaotic; it is often not linear and is certainly not always reproducible. Recently, Thomas, Gonzalez, Pereira,* et al.* have offered a similar argument explaining how the classical Wishnofsky equation involving energy intake minus energy output does not give a consistent pound of weight lost per 3500 kcal removed from the diet over time. These investigators hint that certain variables (e.g., adjustments in BMR (basal metabolic rate),* etc.*) create nonlinear influences that over an extended period of time cause calorically restricted patients to lose somewhat less weight than the Wishnofsky rule would have otherwise predicted [24]. Does this mean we can throw out the First Law of Thermodynamics? Of course not. However, it does hint that something is going on within patients outside the linear confines of our bench lab “measurables”. It is something we need to account for and bring into our discussion. It also suggests that if left unmodified, some of our linear based models for dieting and weight may not be totally accurate.

Using nonlinear models, several predictions can be made about evolving obesity/dietary science and bariatric medicine in general. They can be outlined using what we will call the seven “rules” of nonlinearity. The seven nonlinearity rules are:

1. Expect predictably unpredictable, sudden and continuous change
2. Expect simultaneous but opposite effects from the same agent
3. Expect a repeating order within apparent disorder
4. Expect relatively small effects to have large impacts over time
5. Expect seemingly unrelated processes to affect each other’s outcome
6. Expect resiliency whenever nonlinear processes are in operation
7. Nonlinear analytics always looks for symmetry and reciprocity

## 4. The Tripartite Model and SWEAM

**Figure 1:**
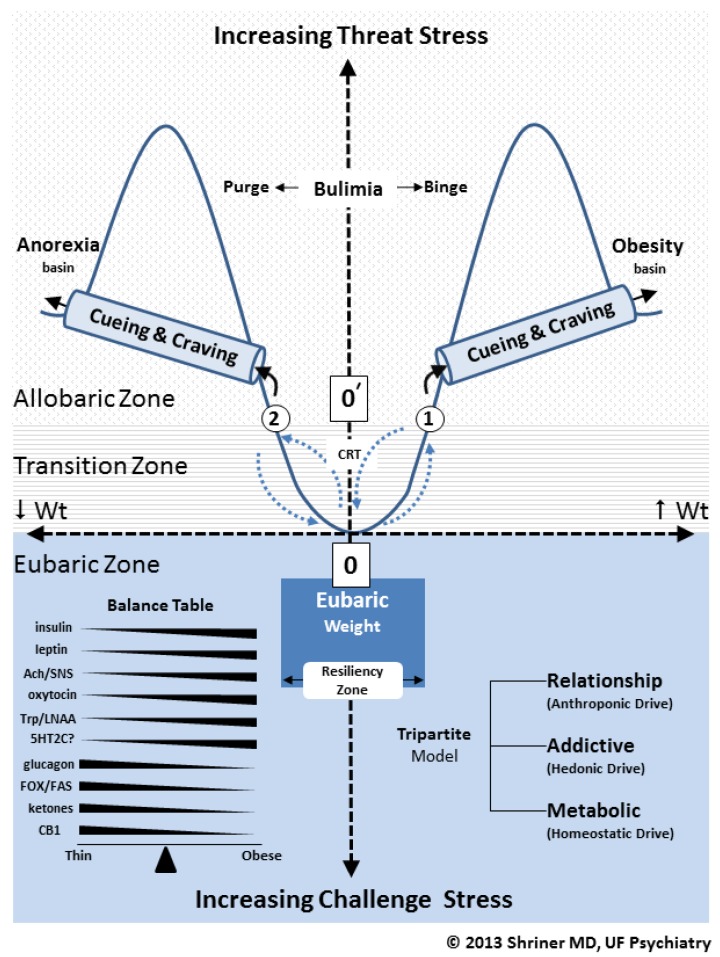
The SWEAM (a stress/weight matrix which stands for: Stress, Weight, Eubaric, Allobaric and Matrix) and Tripartite Models. Abbreviations: Ach (acetylcholine), SNS (sympathetic nervous system), Trp (tryptophan), LNAA (large neutral amino acids), 5HT2C (5-hydroxytryptophan 2 C), FOX (fatty acid oxidation), FAS (fatty acid synthesis), CB1 (endocannabinoid 1).

Shriner has outlined a Tripartite Model for bariatric science [25]. This model is outlined in Figure 1 along the lower right hand corner of the figure. The Tripartite Model consists of three distinct but continuously interacting components or levels. Each level has its own appetitive drive or influence on weight: (1) the metabolic level (homeostatic drive); (2) the addictive level (hedonic drive) and (3) the relationship level (anthroponic drive). The term anthroponic relates to any and all influence that human behavior or psychology has on appetite.

The value of the Tripartite Model is that it outlines three nonlinear, dynamic and continuously interacting inputs to weight that, at any one moment, determine net weight loss or weight gain in human subjects. Food addiction is strategically sandwiched in the middle of this model, between the contributions of metabolic (homeostatic) and relationship (anthroponic) drivers of weight. The advantage of the Tripartite Model is that for the first time, using addictive (hedonic) drivers, we can now explain many of the chaotic and nonlinear reactions that patients have toward food. It is food addiction that helps to explain, in part, the often unstable and unpredictable behaviors of real patients participating in real clinical obesity interventions* versus* those same programs being performed on rats in more linear and controlled bench lab environments. The Tripartite Model (coupled with the SWEAM Model shown in Figure 1) allows us to account for all seven nonlinearity rules we outlined above. Using this new model for obesity and T2DM treatment, we have found that our patients are able to achieve their initial weight management targets and realize more durable weight loss over time.

Examining Figure 1, we have also outlined a weight/stress matrix called SWEAM. This matrix encompasses three distinct but interacting weight environments or zones: (1) eubaric (where “eu” stands for “good” or resilient weight); (2) transitional (involving a normal range of varying daily weights that can border on the pathological or non-resilient) and (3) allobaric (where “allo” stands for “other” or an alternative non-resilient weight) [9]. An example of allobaric weight includes that found in eating disorders such as anorexia, bulimia and binge eating. Alternatively, allobaric weight might also include weight that is being maintained by a pharmaceutical intervention (e.g., patients receiving weight loss medication such as phentermine) or after a surgical intervention (e.g., patients who have undergone gastric bypass surgery). The important point is that although, at any one time, these allobaric patients may appear healthy, they still may be vulnerable to eventual relapse and a reinstitution of their prior unhealthy weight. As we will soon discover, this *vulnerability to relapse* is often driven by hedonic or addictive valences many patients may have toward specific foods and the sequencing of ingestion of these foods (e.g., abstinence followed by bingeing).

The SWEAM outlines two major eating disorders, each being represented as a valley or basin labeled anorexia basin or obesity (*i.e.*, binge eating) basin, respectively. In chaos theory [26], these sorts of basins are called “attractor basins” and as such represent forces in nature that can create reproducible, repeating structures that constitute anomalous eating disorder disease states (see nonlinearity rule 3). The reader should appreciate that as one travels along the “*x*” axis of the SWEAM, from either side of the point of origin labeled “0” (at the center of the matrix), the subject either gains or loses weight. Also note that there is another designation “0ρ” (zero prime) which is the point of origin for all allobaric weights (e.g., circle “1” for allobaric obesity and circle “2” for allobaric pathological fasting).

Again, referring to the SWEAM, as a patient travels along the “*y*” axis of the matrix, he or she either encounters increased threat stress or increased challenge stress. As we stated in nonlinear rule 6, resiliency is a vital component in any successful nonlinear system and this is reflected in the SWEAM. Specifically, only subjects that travel from threat stress toward challenge stress are able to realize resilient weight function over time. The transformation from non-resilient to resilient weight status is shown in Figure 1 by tracing those patients who travel from point “0ρ” to point “0.” Located along this path is the term CRT which stands for cognitive resiliency therapy. This sort of therapy can be actualized by several forms of psychological intervention. However, in all cases, such therapy *must* recalibrate the patient’s allobaric and non-resilient relationships with food (see the Relationship level in the Tripartite Model). Accordingly, this involves a recalibration of anthroponic drives, hence, how patients integrate food resiliently in their relationships with other people.

Resiliency, as a focus of study, is a major factor in human health in all its dimensions [27,28]. In terms of our nonlinear analysis of weight,

*Resiliency may be defined as the ability to take advantage of life’s options in a reciprocal and symmetrical way, so as to avoid the toxic influences of bias.* 

As we just suggested, allobaric patients that take on pathological relationships with food often have a biased or skewed relationship with food, such as we see in the eating disorders of anorexia, bulimia, or binge eating. Eating disordered patients often “limit their options” toward eating since their cognitive biases and valence toward food is infected with alternative meanings, symbolism and attitudes. In its extreme manifestation, food addiction also limits the food addict’s choice of food. Specifically, as we shall outline later, food addicts may be biased toward choosing those foods that stimulate both dopamine and opioids. These foods are often high in sugar or high in sugar/high in fat, but rarely high in fat alone. In this way, we may casually refer to a “chocoholic” or “potato chip binge eater,” but rarely recognize a “Wesson-Oil-o-holic.”

To reiterate, the food addict’s attitude or non-resilient bias toward specific foods may represent a symbolic and usually unconscious substitute for other frustrated or forbidden emotional expressions. For example, anger has been found to be a powerful emotion that can aggravate eating disorders [29]. In this way, eating disorder patients and many food addicts may turn to food in order to quell or derail the expression of more unacceptable (angry) emotions. To be sure, the over-ingestion of food is looked on less negatively by society than the open expression of anger. When this occurs, the food addict (like other forms of addiction) may substitute food for the free and resilient expression of anger and/or a host of other underlying but perceived unacceptable emotions. This is a clear violation of nonlinear rule 7 which demands symmetry and reciprocity of expression. Thus, in the addict, it denies them the ability to cultivate resilient and healthy relationships with the addicting substance. In other words, the food addict fails to understand the reciprocal nature (nonlinear rule 7) of “stuffed anger” and binge eating [30]. Unfortunately, loved ones and society, itself, often misunderstand how sequestered anger can lead to the formation of unhealthy habits, including the abuse of food. Why else would we have come up with the expression “comfort food?” In this way, the compassionate treatment of food addiction (as well as other addictions) may require an improved tolerance and understanding for the healthy expression of a wide variety of emotions such as anger, shame, guilt, empathy,* etc.*, and the value of their nonlinear adjustments. Alternatively, the food addict may fail to understand the symmetrical and reciprocal relationship between high sugar and bingeing. Unless they are compassionately taught, they may never learn that sugar and bingeing have anything to do with addiction, which can then go on to aggravate their type 2 diabetic problems.

In summary, as with other forms of addiction, food and eating can take on a magical quality that biases and lessens the priority of other important choices, sometimes robbing the food addict the capacity to take action (such as putting away toxic and addicting food to prevent the worsening of some underlying illness). In this way, food can take on the same qualities as alcohol or other substances of addiction. The food addict may lose his or her capacity for resilience and freedom to choose food, as food, and not as a substitute for a life of perceived denials and/or defeats. As in other forms of addiction, food exerts an overvalued influence on the food addict’s life, their body and their capacity to enjoy genuine intimacy and love. All clinicians should be aware of these issues as they seek to offer any program of compassionate bariatric care.

Moving on to a discussion of weight, we often find the food addict’s weight no longer reflects a healthy or eubaric weight, but as we just mentioned, takes on an unhealthy or allobaric composition. Thus, food addicts often find themselves operating within the allobaric zone of the SWEAM matrix, leaving them continuously vulnerable to metabolic, addictive, and psychological bias, hence, non-resiliency and relapse.

Seery [31] has clarified that resilience emanates from the way humans negotiate stress from two distinct vantage points: namely, as a *threat* *stress* or as a *challenge stress*. As the SWEAM model suggests, threat stress (see the upper aspect of the “y” axis of Figure 1) increases organismal maladaptation and allobaria over time (hence, it is associated with disordered eating). Alternatively, if the organism is operating under challenge stress (lower aspect of the “y” axis), he or she benefits from greater resilience, suffering considerably less. It is important to recognize that threat stress is often associated with a pro-inflammatory state such as we see in obesity [4]. This is reflected by an elevation of serum cortisol and/or CRF seen in many obese patients, such as we see in patients awaiting bariatric surgery [32]. To make matters worse, Dallman, Pecoraro and la Fleur were able to document that stress related increases in glucocorticoids and their up line signal (*i.e.*, CRF) have a feed forward effect on stimulating the hyper-ingestion of comfort foods such as those high in sugar and/or fat/sugar [33]. This could theoretically create a vicious cycle of stress, binge eating, increased CRF/cortisol and further binge eating leading to more stress,* etc.* As we shall learn, the 2nd (withdrawal) and 3rd (craving) stages of addiction are exacerbated by elevations in CRF [34], which means food addiction withdrawal and craving only heighten and worsen this vicious cycle between stress and an over consumption of high sugar and high sugar/high fat foods. 

Another way of envisioning threat stress is when a problem, rather than being seen as a challenge that motivates change and new adjustments, is seen as a catch-22 which now creates a threat. As it turns out, threat stress can have calamitous effects on patient health. For example, when subjects entertain a problem as a threat stress, their cardiac output and total peripheral arterial resistance both increase, leading to increased blood pressure. Subjects solving this same problem as a challenge stress see their cardiac output increase, but their peripheral arterial resistance drops. In this way, they resiliently avoid suffering increased blood pressure [31].

Given all of the above, it is now clear that the major objective of any successful bariatric (weight management) or eating disorder intervention is to transfer patients from the allobaric zone to the eubaric zone. In other words, assist patients suffering from threat stress (with all its pro-inflammatory, high CRF and stage 2 and 3 addictive features) by helping them to transform this stress into challenge stress. In so doing, they will travel from “0ρ” to “0” down the “y” axis of the SWEAM. During this process they will theoretically decrease their inflammatory stress, gaining greater resiliency and health.

All bariatric clinicians (e.g., dieticians, bariatric physicians, exercise therapists, mindfulness coaches,* etc.*) encounter and deal with threat stress in their patients on an everyday basis. At the University of Florida, we have used a mindfulness CRT (cognitive resiliency therapy) (see Figure 1), called IGS-IT (Information Gathering and Sharing-Information Technology). As outlined elsewhere [35,36], this CRT allows us to utilize a Flow Game learning tool that teaches the patient how to discover and utilize in a nonlinear fashion a set of 5 IGS-rooms within their mind. By learning how to explore these virtual rooms in a resilient and nonlinear fashion, using mindfulness techniques, our patients are able to quit toxic and addictive foods, hold on to more nutritious foods and/or explore creative ways to change their relationship with still other varieties of nourishment. Using the IGS-rooms, patients are able to constructively explore and ventilate their anger, shame, guilt, empathy,* etc.* In this way, using the Tripartite Model, our patients are able to adopt a nonlinear and more resilient relationship with food while at the same time gaining food addiction sobriety.

Before we move on to a discussion of the neurocircuitry of weight, we would like to point out that eubaric (or “good”) weight is a qualitative, not just a quantitative entity. For example, although not shown in Figure 1, bulimics have their own attractor basin. It occupies the area designated within the SWEAM as “bulimia.” This basin was not shown because it would graphically complicate an already busy model. Regardless, the allobaric bulimic patient and the eubaric patient may weigh exactly the same, but the eubaric patient has the qualitative asset of resiliency (something we facilitate with our IGS-rooms CRT) which the untreated bulimic patient does not. Similarly, a post-bariatric surgical patient in the allobaric zone may weigh the exact same weight as one who has undergone CRT and surgery. However, the latter patient has developed resiliency training from CRT and stands much less chance of regaining weight, and/or suffering from post-surgical threat stress. We have treated hundreds of both pre- and post-bariatric surgical patients and most found the Tripartite Model along withIGS-rooms CRT to be extremely useful.

We will now focus on the basic neurometabolic circuitry that creates and sustains the three levels of the Tripartite Model. This will include the principle neurochemical pathways that drive food addiction. We will then analyze the basic processes and behaviors that constitute food addiction, followed by a discussion of suggested treatments.

## 5. Basic Neurometabolic Circuitry of the Tripartite and SWEAM Models

**Figure 2:**
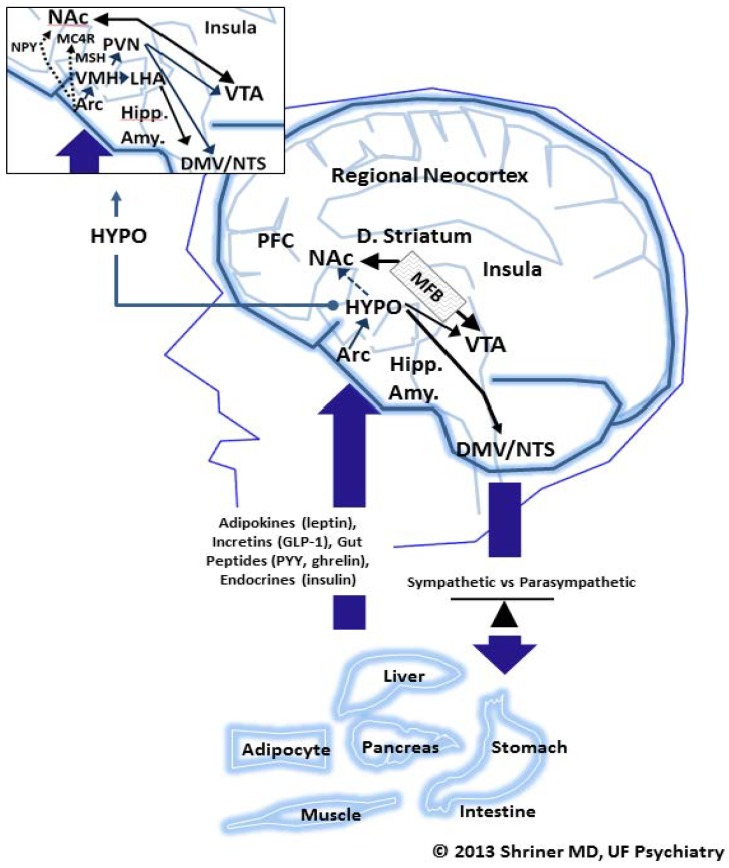
The neurocircuitry of weight. Abbreviations: HYPO (hypothalamus), PFC (Prefrontal Cortex), NAc (nucleus accumbens), D. (dorsal), VTA (ventral tegmental nucleus), Hipp (hippocampus), Amy (amygdala), Arc (arcuate nucleus), DMV (dorsal motor nucleus of vagus), NTS (dorsal motor nucleus of vagus), NPY (neuropeptide Y), MC4R (melanocortin receptor 4), PVN (paraventricular nucleus), VMH (ventromedial hypothalamus), MSH (melanocortin stimulating hormone), LHA (lateral hypothalamus).

Figure 2 diagrams the essential neurocircuitry of weight loss or weight gain driving the functions outlined in the Tripartite and SWEAM models we have just discussed. This neurocircuitry diagram closely follows that proposed by Lustig [37]. Although highly simplified, Figure 2 illustrates the essential neurochemical pathways that lead to food addiction and other pathological adaptations to food.

Beginning along the bottom of Figure 2, specific organs are capable of detecting blood sugar, fatty acids and other components in the blood, which turn on and off key intestinal messengers that are listed inside the large up arrow. Adipocytes, liver, pancreas, stomach, intestine, and muscle are all significant contributors of such messengers, and/or sites of messenger action, as is shown. These neurochemical and neuroendocrine messengers include adipokines (e.g., leptin), incretins, glucagon- like peptide-1 (GLP-1), gut peptides (e.g., ghrelin), and various endocrines; insulin and glucagon, being two principle examples of the latter. Macronutrient stimulated messenger traffic arrives at the arcuate nucleus (Arc) and, in turn, stimulates a set of bicameral (either weight inducing (orexigenic) or weight suppressing (anorexigenic)) responses. For example, when ghrelin production in the stomach is stimulated in response to both appetitive thoughts and actual macronutrients in the gut, it goes on to stimulate orexigenic (increased appetite) responses in the brain at the level of the LHA (lateral hypothalamus), see exploded HYPO (hypothalamus) figure in upper left hand corner of Figure 2. Conversely, insulin (produced in the pancreas) and leptin (produced in the adipocyte) after traveling to the Arc, stimulate anorexia (a decrease in appetite) by their actions on key intermediate chemical agents acting upon the PVN (periventricular nucleus). Insulin and leptin, also, by way of melanocortin 4 receptors (MC4R), exert an anorexigenic influence over the nucleus accumbens (NAc). This nucleus lies outside the hypothalamus, and represents a higher level of central nervous system control over appetite, as shown in Figure 2. As we will soon discuss, the NAc plays a key role in food addiction and the stimulation of appetite. In this way, insulin and leptin may indirectly block food addictive influences at the level of the NAc, causing a loss of appetite. Also note, the PVN goes on to interact with the hindgut (labeled DMV (dorsal motor nucleus of vagus)/NTS (dorsal motor nucleus of vagus)). Via this route, insulin and leptin indirectly stimulate a relative net sympathetic influence back down upon all the peripheral organs outlined in the lower half of Figure 2. This tips the sympathetic* vs.* parasympathetic balance beam shown in Figure 2 to the “left,” favoring fatty acid oxidation and weight loss.

As we just mentioned, the stomach peptide ghrelin essentially reduplicates all that we have seen for insulin and leptin, but in the opposite direction, meaning ghrelin leads to a net increase in appetite. At the level of the Arc, ghrelin stimulates neuropeptide Y (NPY) which exerts an orexigenic (weight inducing) effect on the NAc (opposite to that of MC4R). In addition, as we previously mentioned, ghrelin (and, thus, NPY) stimulate the LHA which exerts a net orexigenic or weight inducing effect on the brain. Finally, just as we saw for the PVN (but now in the opposite direction), the LHA works back on the hind gut (DMV/NTS) to exert a net parasympathetic influence over all the peripheral organs listed in the lower half of Figure 2. This tips the sympathetic *vs.* parasympathetic balance beam to the “right,” favoring fatty acid synthesis and weight gain. It should be noted, that this explanation of PVN and LHA function on appetite is quite simplified. However, the gross assumptions of their net anorexigenic and/or orexigenic influence remain valid.

In summary, as Lustig [37] and others [38] have pointed out, at the level of the adipocyte the relative gain (lipogenesis) or loss (lipolysis) of fat is determined by the net sympathetic (fatty acid oxidation) or parasympathetic (fatty acid synthesis) state that is maintained within peripheral organs such as fat, muscle, liver tissue,* etc.* Again, sympathetic drive/fatty acid oxidation (FOX) favors net weight loss and parasympathetic drive/fatty acid synthesis (FAS) favors net weight gain. For example, the weight loss pharmaceutical reagent phentermine stimulates sympathetic drive, hence, weight loss. Shriner has argued elsewhere that the ultimate aim of any successful bariatric intervention is to drive FOX over FAS [9].

We will now move on to a formal discussion regarding the three principle behaviors (bingeing, withdrawal and craving) that drive food addiction and how these drivers play a key role in explaining the structures labeled “cueing and craving” in Figure 1.

## 6. Food Addiction Equals A Three Step Process

Focusing on Figure 2, the two principle anatomic areas relevant to addiction are: (1) the NAc and (2) the VTA (ventral tegmental area). The two principle neurotransmitters that mediate addiction are dopamine (DA) and mu-opioids [39]. The medial forebrain bundle (labeled MFB in Figure 2) is the key anatomical pathway that connects the NAc with the VTA Regardless if we are talking about cocaine, alcohol, high fructose corn syrup, codeine, marijuana or chocolate, if a substance causes an addictive-like response, it usually deals with the NAc and/or VTA coupled with DA and/or mu-opioids. For example, Pandit, Luijendijk, Vanderschuren,* et al.* were recently able to demonstrate that in rats, NPY acting on the VTA creates the motivation to seek out carbohydrates, whereas NPY acting on the NAc actually mediates consumption [40]. Koob [41] points out that through the MFB, (see Figure 2), mesolimbic DA stimulation plays a central and enabling role in all drugs of abuse. Similarly, he has also argued that all abused drugs decrease reward thresholds (*i.e.*, they increase a sense of reward) during their stimulation phase and increase reward thresholds (*i.e.*, they decrease a sense of reward) during their withdrawal phase. Hoebel, Avena, Bocarsky and Rada [39] have clarified that ventral striatal DA and mu-opioids work in tandem at the level of the NAc to stimulate hedonic consumption of sugar. Kolb and Volkow have outlined three separate stages that must be accounted for in the addiction cycle: (1) the binge/intoxication stage; (2) the withdrawal/ negative affective stage and (3) the preoccupation/anticipation (craving) stage [42].

The binge/intoxication stage, according to Koob [41], begins in either the NAc (e.g., cocaine, opioid influence dependent on DA) or VTA (dopamine dependent stimulation of the opioid receptor). The second or withdrawal/negative affect stage of addiction involves the central nucleus of the amygdala (see Amy in Figure 2), where brain plasticity changes occur that foster and maintain addiction including increased sensitivity for re-exposure to addictive substances. Kolb and Volkow [42] have outlined that the actual affective disturbance called withdrawal may be modulated by increased CRF (corticotropin-releasing factor) As we will argue, CRF is instrumental in effectuating “negative stress” including its negative affective influence on animals and humans suffering from withdrawal. During the withdrawal process, neurotransmitters other than just DA or opioids are also involved (e.g., serotonin, vasopressin, dynorphins, endocannabinoids,* etc.*) [41].

According to Koob [41], the last stage of the addictive cycle is the preoccupation/anticipation (craving) stage. It is during this stage that “cueing” comes into play. As shown in Figure 2, the insula is important in coordinating and involving interoceptive signals coming from the gut, sense organs and the autonomic nervous system and may be significantly involved in cueing (e.g., stimuli that cue or stimulate addictive appetite, like the smell of beer or hot chocolate). Both cueing and craving are centrally important to the process of relapse. Koob has also pointed out that the glutaminergic pathway is vital to cueing and craving through its ability to modify or blunt the normally inhibiting effects that the prefrontal cortex (see PFC in Figure 2) exerts on the NAc and other pro-addictive brain centers [41].

Importantly, Avena, Rada and Hoebel were able to validate in animal models that sugar addicted rats suffered from the same three stages of addiction outlined by Koob including: bingeing, withdrawal and craving, as well as cross sensitization (*i.e.*, rats addicted to sugar also showed increased vulnerability to other addictive substances) [43].

Koob and Volkow [42] have elucidated the ability of CRF to cause negative and adverse feeling states (*i.e.*, withdrawal) which helps to perpetuate the addict’s vulnerability to addictive substances long after they were last abused. This is called *protracted abstinence*. Via this mechanism, if addicts are faced with negative stress there is often an accompanying elevation in CRF which causes the addict to sense withdrawal symptoms. These latter sensations will motivate the addict to seek out and re-engage the original substance of abuse (hence, relapse). In other words, CRF is associated with “feeling bad” and feeling bad is then associated with withdrawal. The addict will re-abuse something he or she thinks will make them feel better. This is how addiction creates its circular and reaffirming hold over the food addict. Again, this whole process is called protracted abstinence.

Finally, as we see in all addictions, the two qualities of “liking” and “wanting” have been demonstrated in food addictions [39]. Berridge [44] has shown that the hotspot for liking is in the shell of the NAc (see Figure 2). It is here that the hedonic and addictive liking aspects of sweetness are kindled by opioids and endocannabinoids. In contrast, Berridge emphasizes that wanting is an entirely different phenomenon involving mesolimbic DA acting on the shell of the NAc, creating salience for addictive thoughts above all others. It is wanting that drives craving, not liking. And, it is the “wanting to feel better” (because of increased CRF) that causes the addict to relapse, including bingeing on chocolate. This is why the food addict will offer, “It’s not that I *like* to eat to feel good (cf. bloating and pain after binge eating), it’s just that I need to eat because I don’t *want* to feel so bad.”

To reiterate, Koob, Volkow and Berridge’s investigations illuminate that the wanting of addiction is associated less with pleasure (liking foods), and more with the negative reinforcement created by their withdrawal. For the food addict, wanting and craving high sugar and high sugar/high fat foods (e.g., ice cream, pizza, McDonald’s fries,* etc.*) are more about trying to prevent the return of negative feelings today, than they are a conscious attempt to deny the calamitous impact these addictive foods will have on the world of tomorrow. 

## 7. The Emerging Science of Food Addiction: Putting It All Together

We can now integrate all the pieces of the nonlinear puzzle affecting food addiction (including the Tripartite and SWEAM Models) in order to construct the most effective bariatric therapy. For the new millennial bariatric clinician, the first issue is to decide where each patient lies within the framework of the SWEAM Model. For example, a patient may be thin or have a fairly low BMI (body mass index) but still be operating within the eubaric zone (see Figure 1) demonstrating adequate resilience. The same may be the case for a moderately obese individual. In fact, slightly overweight geriatric patients may actually live longer than their thinner counterparts [10]. Alternatively, a patient with a normal BMI may be in a state of significant allobaria, such as one suffering from bulimia, where their normal BMI is being sustained by frequent self-induced purging, use of laxatives or periodic unhealthy fasting. Such a patient is decidedly not in a state of eubaria or bariatric resiliency. Similarly, a patient may appear in your office with a newly achieved healthy BMI (e.g., post bariatric surgery or after a course of prescription anorexigenic medication). In Figure 1, they may be at “0ρ” However, if they have not recalibrated any potential anthroponic (human relationship) and/or hedonic (food addiction) drivers, over the next six weeks to six months they may show a sudden re-emergence of binge eating and a rapid return of obesity (*i.e.*, undergo relapse because of protracted abstinence). 

The important point to understand is that food addiction science, envisioned within a nonlinear framework, helps the clinician to prepare both the patient and the clinician for the nonlinear outcomes of dieting, bariatric pharmacy trials or post bariatric surgery. As the seven nonlinear rules on page 3 would suggest, the bariatric clinician needs to anticipate the following:

(1) The sudden re-emergence of either weight gain or weight loss (rule 1).
(2) A dual or paradoxical response of an agent toward weight (rule 2).
(3) A return to a prior exact weight despite all attempts to prevent it (rule 3).
(4) A seemingly minor process has significant impact on weight over time (rule 4).
(5) A seemingly unrelated process cues the return of an exact prior weight (rule 5).
(6) Doing something new or opposite from normal has an impact on weight (rule 6).
(7) Creating a balance and demanding reciprocity in relationships improves weight (rule 7).

Point #1, above, is related to the continuous, dynamic and nonlinear inputs of metabolism, addiction and relationships (the Tripartite Model) that impact net weight from one day to the next, including factors involved in points 2–7.

Point #2 is illustrated by insulin, which has both anorexigenic (inhibits appetite centrally) and orexigenic (stimulates weight gain by increasing fatty acid synthesis, peripherally).

Point #3 is called a topological structure from chaos theory [26] and it results from a repeating confluence of effects or factors on eating. This structure of repeating weight pattern can only be re-engineered differently by undergoing significant revisions in all three levels of the Tripartite Model. Some linear dietician experts have called this recurring structure or weight: set point. However, nonlinear bariatricians understand no weight is truly set, but rather, set point simply represents a repeating weight calibrated to those patients who are living in the allobaric zone. In other words, using nonlinear bariatric programs, patients can escape their “set point” by traveling to the eubaric zone of resilient weight and health.

Point #4 is the result of small effects acting over time that impact large differences. For example, parking your car two rows farther away from the entrance of work can have significant impact on energy expended over time. 

Point #5 has to do with the dramatic impact that the three levels of the Tripartite Model can have on one another. For example, the cue of a family member’s negative comments about an obese patient’s career may trigger threat stress. This, in turn, may stimulate the release of CRF and exacerbate stage 2 and 3 food addiction (which exploit the cueing and craving tunnel outlined in Figure 1). This may trigger a flare of binge eating (wherein the patient enters the cueing and craving tunnel) which facilitates a rapid return of weight gain and forces the patient to return to their former position in the obesity basin. The same may occur for anorexic patients as is shown in Figure 1.

Point #6 deals with the impact of changing the way we handle stress by the thoughts and/or actions we take to overcome it. For example, teaching a patient encountering threat stress to say “why not” instead of “what if” (see CRT mindfulness therapy above) may have a dramatic impact on mitigating perceived negative stress, decreasing fear and CRF, hence, leading to less stage 2 and 3 addiction, less overeating and more weight loss over time.

Point #7 emphasizes the nonlinear impact that seeking and demanding symmetry and reciprocity has on fostering the emergence of resiliency. For example, without totally alienating him or her, requesting that your boss try and give you some forewarning about staying late after work, may have a dramatically soothing effect on mitigating threat stress, unconscious anger, shame and guilt, increased CRF and binge eating.

## 8. Suggested Treatments for Weight that Incorporate Food Addiction Management 

The first step in treating food addiction is making the diagnosis. Gearhardt,* et al.* have introduced the Yale Food Addiction Scale which can easily be administered to both adults and children/adolescents [19,20]. Their scale addresses addictive criteria including food tolerance, food withdrawal and the seeking and abusing of food despite knowledge of its dangerous consequences. Shriner [9] has offered, “Food addiction represents a pervasive and enduring pattern of both food perception (how we view and feel about food) and food related behavior (how we go at procuring and ingesting food) which biases our relationship with food in harmful, non-resilient and unhealthy ways.” Given all of the nonlinear complexities of natural processes [26], including those that effect and affect food addiction, patients suffering from disordered eating (including obesity or diabesity) should be evaluated by keeping both the Tripartite and SWEAM Models in mind.

We will now outline some of the major divisions of obesity intervention that include and integrate nonlinear models and what we have just learned about effective weight management.

### 8.1. Metabolic Interventions: Macronutrient and Pharmacy Prescriptions

Before we formally comment on macronutrients and pharmacy, the reader should briefly inspect the “balance table” located along the lower left hand corner of Figure 1. This table outlines some gross vectors of change in various neurotransmitters, endocrines, gut peptides,* etc.*, as the patient moves from weight loss to weight gain or *vice versa*.

As we mentioned previously, the central aim in the dietary or macronutrient intervention of obesity and diabesity is to understand the importance of nonlinear factors that ultimately (as outlined in Figure 2) favor net FOX over FAS. The ratio of FOX to FAS is outlined in the balance table in Figure 1. As one can see from this table, lower weights are often associated with higher FOX, lower insulin, higher glucagon, increased ketones, *etc.* Those diets that can instrument some of these strategic nonlinear metabolic changes will most likely lead to more successful weight loss. In 2008, the American Diabetic Association approved low carbohydrate diets [45] for diabetics and the American Heart Association has urged that patients at risk for heart disease significantly curtail their overconsumption of sugar [46]. Haimoto, Sasakabe, Wakai and Umegaki, have offered that even a 45% carbohydrate diet led to greater reduction in hemoglobin A1c (compared to higher carbohydrate diets) in patients suffering from T2DM [47]. Shai,* et al.* reported in the New England Journal of Medicine that low carbohydrate and Mediterranean Diets beat low fat diets for weight loss and lipid changes for up to two years [48]. Kusher and Doerfler outline how low carbohydrate, high protein diets may be an effective choice for weight loss, enhanced satiety and improved metabolic parameters [49]. Neilsen and Joensson have reported that low carbohydrate diets (*i.e.*, 20% carbs) in T2DM patients remain superior to higher carbohydrate diets (*i.e.*, 55%–60% carbs) all the way out to 44 months [50]. Finally, Mithieux,* et al.* have shown how higher protein diets can stimulate at the level of the small intestine an anorexigenic signal back to the brain leading to an earlier and higher satiety rate per unit of caloric intake [51].

Given the above findings, we found in our Living with Food obesity program that low carbohydrate (~45–60 g per day) coupled with higher protein diets were highly effective and well tolerated. We preferred the superior aspects of whey protein powder [52] in the form of Greek yogurt protein shakes. Finally, given Lustig’s critical work [12] on the marked adverse effects of high fructose corn syrup on diabesity, we strongly recommended that our patients limit their ingestion of this substance.

Bariatric pharmacy continues to progress and the balance table in Figure 1 outlines much of its rationale. Edge and Gold have recently published an investigational paper on the potential role of addiction based pharmacotherapy in the treatment of eating disorders, qualifying the limitations of specific drugs for long term use [53]. Rosen and Aronne have updated some of the latest Food and Drug Administration (FDA) approved anti-obesity agents that are currently available [54]. Of these, phentermine may work by stimulating SNS predominance resulting in FOX > FAS, hence, weight loss (see balance table). Qsymia (phentermine hydrochloride + topiramate) works similarly, with the added benefit of the anticonvulsant topiramate whose exact mechanism of action is yet to be established [55]. Perhaps, since it antagonizes glutamate receptors (whose involvement in stage 3 addiction we outlined above), topiramate may interfere with craving and cueing. Contrave (naltrexone + topiramate) will hopefully soon be approved by the FDA after results of the Light Study are concluded [56]. Obviously, by way of its modulation of opioid signaling, naltrexone may help to lessen the liking aspects of addictive foods and their impact on weight.

Finally, given the ability of endocannabinoids (see CB1 in Figure 1) to stimulate DA [57] which can effectuate orexigenic responses in the brain, the usage of marijuana should probably be strongly discouraged in those patients seeking meaningful assistance with either weight loss management or diabesity medical intervention. In addition, given the central importance of *de novo* production of dopamine in suppressing weight gain through its sympathetic contributions to sympathetic/parasympathetic balance (see Figure 2), the withdrawal of dopamine based anorexigenic agents can result in a significant regain in weight [14]. In addition, such withdrawal can theoretically exacerbate CRF release, causing food addicts to relapse, given CRF’s role in the processes of stage 2 and stage 3 addiction. Decidedly, this may be a potential downside to the routine use of sympathomimetic agents such as phentermine in weight management programs. It underscores why these agents should arguably be employed only for a relatively short period, using other non-drug therapies to eventually ensure more durable and resilient weight loss over time.

### 8.2. Addictive Interventions: Avoiding Addictive Foods, Valuing Fellowship and Relapse Prevention

Hoebel,* et al.* were able to show in rats that *sugar*, but not fat, was capable of producing the classical symptoms of drug withdrawal [39]. Of relevance to binge eating, Avena, Rada and Hoebel demonstrated that intermittent (but not continuous) intake of large quantities of highly palatable food (high sugar or high sugar/high fat) followed by abstinence, sets up binge eating in rats, down line [43]. Overall, from these and other studies, food addiction science is beginning to suggest that foods higher in dopamine and mu-opioid agonism (*i.e.*, ones that stimulate the production of dopamine or opioids), such as high sugar colas, desserts and cafeteria style foods, may constitute the best nutritive candidates for food addiction. At this point, however, this is only speculation. In addition, the sequence or timing of ingestion may also be of importance, wherein, intermittent abstinence (the sudden stoppage of ingestion of addictive foods) may create a ripe environment for binge eating at a later period in time. This means that diets should encourage continuous eating patterns of ingestion and not periodic abstinence (e.g., “I will never eat chocolate again, since I just gorged myself on a bag full of those little devils!”). Finally, in order to achieve and maintain food sobriety over time, social networking and fellowship appear to be of tremendous value [58]. In our University of Florida Living with Food program, we found that those patients who attended groups and participated in aftercare (*i.e.*, patient run groups) achieved the greatest and most durable weight loss at the time of follow up.

### 8.3. Relationship Interventions: The Power of Mindfulness Therapy

As we alluded to earlier, CRT or cognitive resiliency therapy (see Figure 1) is instrumental to the process of teaching patients how to lead themselves from the allobaric zone into the eubaric zone of the SWEAM. Many forms of such therapy exist, but the one we have employed at the University of Florida (IGS-rooms) [9], has uncovered five principle Flow Emotions (guilt, power, empathy, passion and anger). It is our hypothesis that many, if not all, of these emotions may play specific roles in fostering obesity and other disorders of eating as suggested by studies involving anger [29], guilt [59,60,61] and shame [59,62] in patients suffering from eating disorders and/or obesity. IGS-room CRT allows patients to discover their default favorite flow emotion (called their home room) and then teaches them how to use all five Flow Emotions to construct QHC-food (Quit, Hold and Change) contracts. These food contracts, coupled with CRT, give patients the tools to overcome food addiction through time. When these contracts are employed in the everyday lives of patients, they develop increased mastery over stress and the biases that formerly drove them into addictive relations with food.

Patients in the Living with Food program learned how to cook less addictive and less diabesigenic meals via our food kitchen and group discussion formats. As stated above, they learned the value of fellowship support networks and attended aftercare patient run groups. They learned how to more effectively deal with, express, and reintegrate the five Flow Emotions (especially anger, guilt and empathy). This defused their need to use food as a substitute for not feeling valued by others. Throughout, they learned how to live with the seven nonlinear rules we have identified in this paper, which added significant resiliency to their lives.

Lastly, our obesity and type 2 diabetes prevention programs have sought to emphasize the value of exercise in the treatment of obesity and diabesity. Exercise, which attenuates ghrelin [63], also increases lipoprotein lipase mediated triglyceride clearance, activates AMPK (AMP-activated protein kinase) (which increases fatty acid oxidation), as well as GLUT 4 insulin receptor activity, improving glucose clearance from the blood [38]. With all these and other potential benefits in mind, exercise and (when indicated) physical therapy are indispensable to successful weight loss and the mitigation of diabesity.

## 9. Qualifications, Alternative Viewpoints and Caveats

Throughout this article we have focused on the concept of food addiction as an extension of other forms of addiction, including the mechanisms of addictive process (bingeing, withdrawal and craving). We have pointed out that in animals models, clearly certain foods are more addictive than others, with highly palatable substances such as sugar and cafeteria style foods appearing to be nutrients of choice for addictive response in these animal models. In terms of the relevance of these claims to humans, however, there remain important unanswered questions and caveats. Specifically, although Gearhardt,* et al.* [19] have shown definite bingeing, craving and continued ingestion of food despite known harmful consequences (as well as many other features of the DSM-5 criteria for addiction) in their food addiction (YFAS or Yale Food Addiction Scale) assessment scale, the empirical evidence supporting actual food withdrawal seems in need of either further study [19] or, according to some experts, is lacking altogether [64]. Thus, although substances such as sugar and high fat/high sugar foods have been found to be addictive in animal models, their equivalent addictive value in humans is still under active debate and in need of further investigation [65]. To be sure, several authors have contested the assumption that the abuse of food should be implicated alongside other forms of addiction, or that food is necessarily addicting. Their arguments, of course, have merit. For example, Ziauddeen, Farooqi and Fletcher [64] rebut the value of food addiction, in general, while Meule [66] offers instances where the YFAS failed to differentiate food addiction from BMI. Interestingly, in the latter instance, Meule offers possible nonlinear solutions for these apparent inconsistencies. It should also be noted that the DSM-5 [67] failed to include food addiction as a recognized mental disorder. It could also be argued that rather than involving a truly addictive phenomenon, the hyper-ingestion of food (such as we often see with sugar) may simply represent a manifestation of “hedonia”. Supporters of food addiction science, however, address this argument in part by pointing out that many patients who identify themselves as food addicts do not necessarily like the foods they abuse, especially during their bingeing episodes. In other words, they may report less hedonic or liking valences toward food and more of a desire to avoid negative feelings or not wanting to feel bad when they are deprived access to their favorite foods of abuse along the lines outlined by Berridge [44] and Koob [41]. It has been our experience in the Living with Food Program at the University of Florida that many of our patients disliked the very foods they tended to abuse, but felt compelled to continue to abuse them. In an attempt to account for this phenomenon the Tripartite Model includes the designation of addiction, instead of confining this level to the simple designation of hedonia, even though such addiction may involve at least initially hedonic drivers, again, as described by Berridge and Koob.

Certainly, there remains considerable work yet to be done in terms of the study of the abuse of food and/or food addiction for it to achieve the same degree of validity granted to the study of more traditional addictive disorders (*i.e.*, stimulant abuse, alcoholism,* etc.*). Notwithstanding these caveats, however, the value of food addiction science as an area of further study remains intriguing, if not compelling. Food addiction science continues to offer us potential clarity in terms of explaining the complex and often chaotic and inconsistent responses that patients may have toward food over time. This same science may also assist clinicians to identify the most effective therapies to deal with these same complex patients. Finally, it should be pointed out that the social environment in which food is introduced to the patient plays an important role in the phenotypic expression of disordered eating. For example, Brownell and his colleagues [68,69,70,71] have outlined how important food advertisement and the media’s manipulation of food may have on helping to drive the hyper-ingestion of specific macronutrients that many investigators argue may become addicting over time.

## 10. Conclusions

Within this review of food addiction, we have outlined some of the more important challenges and opportunities that lie ahead in the treatment of obesity and diabesity. As an emerging nonlinear science, food addiction will play a vital role in facilitating the discovery of new and more effective nutritional and pharmaceutical therapies. We have offered several models that will hopefully assist others in their interventions with obese and diabesity patients. These models demonstrate the complexity, as well as advantage, of continuously and dynamically integrating metabolic, addictive, and human relational factors in obesity management. It may be that Hippocrates was quite correct. Dieticians, assisted by other bariatric clinicians, may already have at their disposal the ultimate pharmaceutical to combat obesity, namely, food itself.
